# Impact of earplugs and eye mask on sleep in critically ill patients: a prospective randomized study

**DOI:** 10.1186/s13054-017-1865-0

**Published:** 2017-11-21

**Authors:** Alexandre Demoule, Serge Carreira, Sophie Lavault, Olivier Pallanca, Elise Morawiec, Julien Mayaux, Isabelle Arnulf, Thomas Similowski

**Affiliations:** 1 Neurophysiologie respiratoire expérimentale et clinique, Sorbonne Universités, UPMC Université Paris 06, INSERM, UMRS1158, Paris, France; 2 Service de Pneumologie et Réanimation Médicale (Département “R3S”), Assistance Publique – Hôpitaux de Paris, Groupe Hospitalier Pitié-Salpêtrière Charles Foix, 47-83 boulevard de l’Hôpital, 75651 Paris, Cedex 13 France; 3 Assistance Publique – Hôpitaux de Paris, Groupe Hospitalier Pitié-Salpêtrière Charles Foix, Service des pathologies du sommeil, Paris, France; 4 Institut du cerveau et de la moelle, Sorbonne Universités, UPMC Université Paris 06, Paris, France

**Keywords:** Sleep, Intensive care, Earplugs, Eye mask, Delirium, Polysomnography

## Abstract

**Background:**

Poor sleep is common in intensive care unit (ICU) patients, where environmental factors contribute to reduce and fragment sleep. The objective of this study was to evaluate the impact of earplugs and eye mask on sleep architecture in ICU patients.

**Methods:**

A single-center randomized controlled trial of 64 ICU patients was conducted from July 2012 to December 2013. Patients were randomly assigned to sleep with or without earplugs and an eye mask from inclusion until ICU discharge. Polysomnography was performed on the first day and night following inclusion. The primary outcome was the proportion of stage N3 sleep over total sleep time. Secondary outcomes were other descriptors of sleep and major outcome variables.

**Results:**

In the intervention group, nine (30%) patients did not wear earplugs all night long. The proportion of N3 sleep was 21 [7–28]% in the intervention group and 11 [3–23]% in the control group (*p* = 0.09). The duration of N3 sleep was higher among the patients in the intervention group who wore earplugs all night long than in the control group (74 [32–106] vs. 31 [7–76] minutes, *p* = 0.039). The number of prolonged awakenings was smaller in the intervention group (21 [19–26] vs. 31 [21–47] in the control group, *p* = 0.02). No significant difference was observed between the two groups in terms of clinical outcome variables.

**Conclusions:**

Earplugs and eye mask reduce long awakenings and increase N3 duration when they are well tolerated.

**Trial registration:**

ClinicalTrials.gov, NCT02292134. Registered on 21 Nov 2013.

**Electronic supplementary material:**

The online version of this article (doi:10.1186/s13054-017-1865-0) contains supplementary material, which is available to authorized users.

## Background

Over the past decade, a large body of literature has raised the major issue of sleep disturbances in critically ill patients [1, 2]. Sleep is reduced, fragmented, does not follow the regular circadian rhythm, and contains increased N1-N2 stages, to the detriment of N3 and rapid eye movement (REM) sleep. Importantly, N3 and REM sleep play a critical role in many physiologic functions, including the central nervous, cardiovascular, endocrine, respiratory, and immune systems. The deleterious consequences of poor sleep in patients admitted to the intensive care unit (ICU) are becoming increasingly clear [1]. Poor sleep is a risk factor for delirium [3], noninvasive ventilation failure [4], and an intrinsic ICU stressor for patients [5], which may in turn participate in the mechanisms of posttraumatic stress disorder [6]. The impact of poor sleep on immune function, metabolism, length of mechanical ventilation, and post-ICU quality of life is also suspected but has not been clearly demonstrated [7]. Sleep improvement has therefore become a goal of care in the ICU [8].

The multiple mechanisms responsible for altered sleep include environmental factors such as noise and light, including those related to human interventions [9]. However, the reduction of noise and light during the night, although theoretically feasible, is not easy to achieve in the ICU, because a high level of human activities in the ICU during the night may be required by the condition of admitted patients or the admission of new patients. In addition, for safety reasons, the noise level of alarms cannot always be turned off or even lowered [10]. An alternative strategy would be to protect patients individually against noise and light by means of earplugs and an eye mask. Previous reports have suggested that this strategy could increase sleep quality in healthy subjects submitted to a level of noise and light encountered in an ICU [11] and in patients sleeping in the postanesthesia care unit [12]. However, the impact of earplugs and an eye mask on sleep quality has not previously been evaluated in ICU patients by polysomnography.

We therefore conducted a randomized controlled trial to determine the efficacy of this strategy on the basis of the hypothesis that earplugs and an eye mask would improve sleep quality during the first night following the initiation of the intervention in critically ill patients, as measured by the proportion of N3 sleep, also known as non-REM sleep stages 3 and 4 or slow-wave sleep. We selected this criterion because N3 sleep is not only thought to be the most “restorative” sleep stage [13–15] but also is involved in the pathogenesis of metabolic and cardiovascular diseases [16, 17] and is noticeably reduced in ICU patients [9, 18, 19].

## Methods

Human research ethics committee approval for the study was provided by the Comité de Protection des Personnes - Ile de France 6. Patients or their next of kin gave informed consent. Data were collected from July 2012 to December 2013.

### Site

This study was conducted in a 16-bed adult general ICU within a 1600-bed hospital in Paris. The ICU is arranged as two rows, one comprising ten rooms and one comprising six rooms, with one patient per room. Each room has the same organizational layout, with one door leading to the common hallway and one wall containing a large window facing either north or south. Three intensivists are responsible for the management of all patients from 8:30 a.m. to 7:00 p.m., and one intensivist covers the night. Ward rounds are conducted by the intensivists three times per day. The nurse-to-patient ratio is between 1:2.5 and 1:3, depending on the intensity of care. Patient care activities occur according to defined schedules.

Sleep quality is an important aspect of care in our unit, and general rules are routinely applied to promote nighttime sleep and to avoid sleep disruption. Lights are generally turned off in rooms and dimmed in corridors at 10:00 p.m. Patient televisions are turned off, in-room alarms are minimized, and care activities are grouped [8]. In addition, window blinds are raised during day, and mobilization is encouraged in order to promote normal circadian rhythms.

### Patients

Patients meeting the following criteria were included: (1) no sedation for > 24 h, (2) sedation level < 3 on the Ramsay Sedation Scale, (3) expected remaining ICU stay > 48 h, and (4) morphine < 0.01 mg/kg/minute and norepinephrine < 0.3 μg/kg/minute. Exclusion criteria included a history of sleep disorders such as sleep-related breathing disorders, insomnias, or sleep movement disorders; psychiatric illness requiring chronic medication; a known diagnosis of central neurological impairment; liver disease with encephalopathy; uncontrolled sepsis; severe hearing impairment; or blindness. Patients aged < 18 years were also excluded.

### Study design

Randomization was performed using a computer-generated sequence provided through a website. Patients were allocated to one of two different groups: The control group received routine care during the night, and the intervention group received routine care plus an eye mask (Slaapmasker Schlafmaske, Stuttgart, Germany) and earplugs (Samurai, Vandeputte Group, Oosterhout, The Netherlands). The intervention was applied every night at 10:00 p.m. until 8:00 a.m. from inclusion until ICU discharge. Trained nurses placed the devices.

### Data collection and analysis

Simplified Acute Physiology Score II and Charlson comorbidity index score [20] were calculated on admission. Physiologic data such as heart rate, arterial blood pressure, respiratory rate, and temperature were also recorded, as were blood gases. The patient’s self-reported comfort and sleep quality were assessed daily using a simplified visual analogue scale (VAS; 10 cm horizontally) from zero for worst possible comfort or sleep quality to 10 for best possible comfort or sleep quality. The presence of delirium was evaluated once daily by a nurse using the Confusion Assessment Method for the ICU [21].

Polysomnography was performed on the day of inclusion using a portable device (Dream®; MEDATEC, Anderlecht, Belgium). Recording lasted 18 h, starting at 2:00 p.m. and ending at 8:00 a.m. Electroencephalography (EEG) with electrodes placed at O1/A2 and C4/A1 according to the international 10–20 system, electromyography (electrodes located on the levator menti muscle), electrooculography (left superior canthus, right inferior canthus), electrocardiography, and pulse oximetry were recorded. No video was used. Sleep recordings were visually scored by a sleep specialist physician blinded to the group using international criteria [22].

Ambient sound was continuously recorded until ICU discharge at the level of the patient’s head with a portable sound meter (SL407760; Littoclime, Caen, France). Time with lights on was continuously recorded until ICU discharge by a camera pointing at the ceiling of the patient’s room. Number and length of nurse interventions during the night (from 10:00 p.m. to 8:00 a.m.) were recorded until ICU discharge by bedside nurses. Compliance with earplug and eye mask use was recorded by bedside nurses.

At ICU discharge, using a VAS, patients self-assessed overall sleep quality and comfort during their stay. At ICU discharge and at day 90 following randomization, patients were assessed using the Hospital Anxiety and Depression Scale (HADS) [23, 24]. In addition, patients were assessed at day 90 for sleep quality using the Pittsburgh Sleep Quality Index and for posttraumatic stress disorder-related symptoms using the Impact of Event Scale–Revised (IES-R) [25]. Data were recorded on electronic case report forms powered by a data manager (CleanWEB™; Telemedicine Technologies, Boulogne-Billancourt, France).

### Statistical analysis

The primary outcome variable was the proportion of total sleep time spent in N3 sleep during the first day and night (from 2:00 p.m. to 8:00 a.m.) following inclusion. Secondary endpoints were sleep quality, REM sleep, sleep efficiency (number of minutes of sleep divided by the number of minutes recorded), index and number of arousals, short awakenings (awakenings lasting < 1 minute) and prolonged awakenings (awakenings lasting > 1 minute), and awakenings during the first day and night following inclusion. Other secondary endpoints were sleep quality measured with a VAS sleep scale; presence of delirium, anxiety, and depression on ICU discharge and on day 90; ICU and hospital length of stay and mortality; presence of posttraumatic stress disorder; and sleep quality on day 90.

On the basis of previous reports of sleep architecture in the ICU [9, 18, 19], we estimated the mean N3 proportion of total sleep time in patients comparable to our study population to be 2.9% with an SD of 3.3%. We assumed that the N3 proportion would increase to 5.8% in patients receiving routine care plus eye mask and earplugs, but that it would remain at 2.9% in other patients. The effect size between these two means was 0.879 on a 0–1 scale. Sample size calculations showed that 25 patients per group would provide 80% power at a two-sided level of 0.05 to detect an N3 increase. With an estimated 25% polysomnography failure rate, the final calculated sample size was 64 patients.

Continuous variables are described using the median and IQR. Categorical variables are described using frequency and percentage. Statistics were performed with SAS version 9.3 software (SAS Institute Inc., Cary, NC, USA).

Differences between groups were assessed with the Mann-Whitney *U* test for continuous variables and the χ^2^ test for categorical variables. The primary analysis was done on the basis of the intention-to-treat principle. Because we further noticed that a substantial number of patients did not follow the intervention (i.e., did not wear earplugs all night long), we decided to perform a secondary post hoc analysis to compare patients who actually wore earplugs all night long in the control and intervention groups.

## Results

### Enrollment, study population, and sleep recordings

**Fig. 1:**
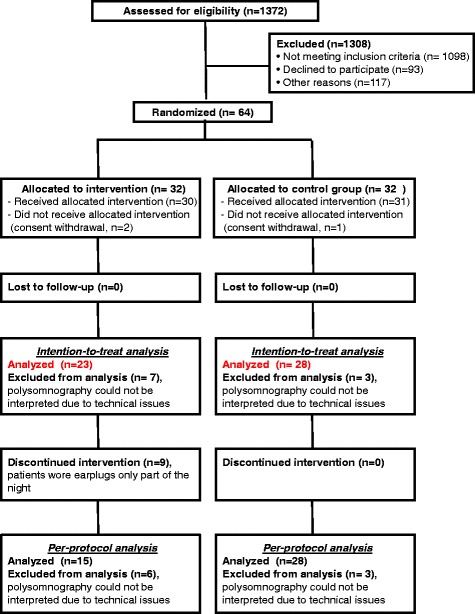
Study flowchart

We prospectively screened patients between July 2011 and December 2013 and 64 patients were enrolled, 32 in each group (Fig. 1). Three patients withdrew consent after randomization, two in the intervention group and one in the control group.

During the first night following inclusion, 21 patients wore earplugs all night long in the intervention group, and 18 of these patients wore their eye mask. Nine patients wore earplugs only part of the night, and one patient wore the eye mask alone. The main reasons for refusing to wear earplugs or the eye mask were discomfort and anxiety.

Baseline patient characteristics are displayed in Table 1. The two study groups were balanced at baseline in terms of age, comorbidities, severity of illness, reason for ICU admission, days of sedation and time from end of sedation to inclusion, length of ICU stay prior to inclusion, physiologic variables, and ventilation and oxygenation parameters (Additional file 1: Table S1). Nurse interventions tended to be lower in the intervention group, and noise level was similar in the two groups (Additional file 2: Table S2).

**Table 1:** Main characteristics of included patients

	Control group (*n* = 31)	Intervention group (*n* = 30)	*p* Value
Patient characteristics			
Age, years	65 (58–74)	64 (54–74)	0.99
Sex, male, *n* (%*)*	18 (58)	20 (67)	0.48
SAPS II score	45 (27–65)	42 (26–60)	0.60
Charlson comorbidity index score	4 (3–6)	4 (2–5)	0.30
Main reasons for ICU admission, *n* (%)			
Acute respiratory failure	23 (74)	20 (67)	0.71
Pneumonia/pleural effusion	11	8	
Chronic respiratory disease	4	7	
Cardiogenic pulmonary edema	5	3	
Neuromuscular disease	2	0	
Vascular disease	1	2	
Postoperative care/trauma, *n* (%)	3 (10)	4 (13)	0.96
Metabolic, *n* (%)	3 (10)	4 (13)	0.96
Nonrespiratory sepsis, *n* (%)	2 (6)	2 (7)	0.63
Prior to inclusion			
Days of sedation	3 (2–8)	4 (2–7)	0.30
Time from end of sedation to inclusion, days	3 (0–4)	1 (0–4)	0.49
On inclusion			
RASS	0 (0–0)	0 (0–0)	0.19
Comfort score, VAS	60 (50–80)	70 (50–80)	0.92
Physiologic variables			
Temperature, °C	37.1 (36.9–37.4)	37.1 (36.2–37.6)	0.66
Heart rate, beats/minute	98 (83–116)	86 (76–108)	0.29
Systolic blood pressure, mmHg	115 (106–140)	130 (121–134)	0.19
Respiratory rate, breaths/minute	22 (20–25)	19 (16–25)	0.18
Analgesic treatment, *n* (%)	17 (55)	11 (37)	0.15
Mechanical ventilation	6 (19)	5 (17)	0.35
Invasive ventilation, *n* (%)	6 (19)	3 (10)	
Noninvasive ventilation, *n* (%)	0	2 (7)	

*RASS* Richmond Agitation-Sedation Scale, *SAPS II* Simplified Acute Physiology Score II, *ICU* Intensive care unit, *VAS* Visual analogue scale (0 = maximal discomfort, 100 = maximal comfort)

Results are expressed as median (interquartile range) or frequency (%)

### Primary outcome: sleep architecture

Polysomnography could not be scored accurately, owing to poor signal quality in seven patients in the intervention group and three patients in the control group. These patients were included in the analysis but did not contribute to the primary outcome. Data for the primary outcome variable were subsequently collected for 23 patients in the intervention group and 28 patients in the control group.

Sleep measurements are detailed in Table 2. The N3 proportion was not different between the two groups (21 [7–28]% in the intervention group vs. 11 [3–23]% in the control group, *p* = 0.09). Prolonged awakenings were less frequent in the intervention group (21 [19–26] %) than in the control group (31 [21–47] %, *p* = 0.02). Total sleep time during the 18-h recording and during nighttime, REM sleep time and percentage, sleep efficiency, and number of short awakenings (< 1 minute) and arousals were not significantly different between the two groups. Sleep quality following the first night after inclusion was similar in the two groups.

**Table 2:** Main sleep characteristics of the patients in whom polysomnography could be accurately scored

	Control group (*n* = 28)	Intervention group (*n* = 23)	*p* Value
Total sleep time per 18 h^a^, minutes	301 (229–398)	290 (146–410)	0.91
Total sleep time during nighttime^b^, minutes	274 (177–329)	286 (120–392)	0.77
N1 Stage, minutes	29 (17–59.5)	29 (6–50)	0.42
N2 Stage, minutes	182 (102–229)	146 (84–223)	0.32
N3 Stage, minutes	31 (7–69)	58 (24–86)	0.16
N3 stage, % of total sleep time	11 [3–23]	21 [7–28]	0.09
REM sleep, minutes	32 (6–48)	35 (9–60)	0.64
Sleep efficiency^a^, % per 18 h	27 (21–38)	26 (14–42)	0.72
Short awakenings (<1 minute), *n*	11 (5–20)	8 (3–13)	0.23
Prolonged awakenings (>1 minute), *n*	31 (21–47)	21 (19–26)	0.02
Awakenings and arousals, *n*/TST	26 (13–46)	24 (15–29)	0.39
Self-assessed sleep quality, VAS	50 (32–70)	50 (40–60)	0.81

*REM* Rapid eye movement, *TST* Total sleep time, *VAS* Visual analogue scale from zero (poor sleep quality) to 100 (excellent sleep quality)

^a^Total recording period is from 2:00 p.m. to 8:00 a.m.

^b^Nighttime is from 10:00 p.m. to 8:00 a.m.

We compared the 31 patients in the control group with the 21 patients in the intervention group who actually wore earplugs all night long in a per-protocol analysis. Among them, polysomnography could be scored in 15 patients (Additional file 3: Table S3). N3 sleep time was higher and prolonged awakenings were less frequent in intervention group patients who wore earplugs all night long than in the control group patients.

### Secondary outcomes

In the intervention group, evaluation was performed at ICU discharge in 23 patients, at hospital discharge in 22 patients, and at day 90 in 16 patients. In the control group, evaluation was performed at ICU discharge in 22 patients, at hospital discharge in 21 patients, and at day 90 in 18 patients (Additional file 4: Figure S1).

Table 3 displays the main outcome variables. No significant difference was observed among the intervention and control groups in terms of sleep quality and presence of delirium during the ICU stay. On ICU discharge, VAS-assessed sleep quality and comfort throughout the ICU stay, anxiety and depression, ICU length of stay and mortality, and hospital mortality and length of stay were not significantly different between the two groups. On day 90, anxiety and depression as assessed using the HADS, sleep quality as assessed using the Pittsburgh Sleep Quality Index, and IES-R were not significantly different between the two groups.

**Table 3:** Main outcomes at intensive care unit discharge, hospital discharge, and day 90 follow-up

	Control group	Intervention group	*p* Value
At ICU discharge^a^	*n* = 22	*n* = 23	
Self-assessed sleep quality, VAS	60 (25–80)	70 (50–70)	0.63
Self-assessed comfort score, VAS	70 (50–80)	70 (70–80)	0.68
Anxiety score, HADS	9 (6–11)	8 (6–10)	0.66
Depression score, HADS	8 (4–9)	4.5 (2–9)	0.25
Delirium, *n* (%)	2 (6)	2 (7)	1
ICU length of stay, days	7 (5–26)	7 (4–11)	0.18
ICU mortality, *n* (%)	4 (13)	3 (10)	0.99
At hospital discharge^b^	*n* = 21	*n* = 22	
Hospital length of stay, days	26 (14–86)	24 (12–47)	0.76
Hospital mortality, *n* (%)	4 (13)	3 (10)	0.76
At day 90 follow-up^c^	*n* = 18	*n* = 16	
Anxiety score, HADS	6 (4–12)	8 (4–11)	0.69
Depression score, HADS	6 (2–9)	6 (3–12)	0.63
Pittsburgh Sleep Quality Index	5 (5–8)	8 (5–11)	0.25
Impact of Event Scale–Revised	16 (9–27)	11 (5–18)	0.15

*ICU* Intensive care unit, *VAS* Visual analogue scale from zero (poor) to 100 (excellent), *HADS*, Hospital Anxiety and Depression Scale

Results are expressed as median (IQR) or frequency (%)

^a^Six patients died in the ICU, and ten were unable to answer questionnaires at ICU discharge. Consequently, evaluation at ICU discharge was performed in 45 patients

^b^Two patients died in the hospital after ICU discharge. Consequently, evaluation at hospital discharge was performed in 43 patients

^c^Seven patients lost to follow-up between hospital discharge and day 90 and two were unable to answer questionnaires. Consequently, evaluation at day 90 was performed in 34 patients

## Discussion

The main findings of this study can be summarized as follows. Earplugs and an eye mask applied from awaking following interruption of sedation and until ICU discharge (1) failed to significantly increase the proportion of N3 sleep on the first night following inclusion, but significantly decreased the number of prolonged awakenings; (2) were poorly tolerated, but may increase the duration of N3 sleep in patients who tolerate them; and (3) had no impact on outcome.

This study demonstrates that sleep was severely altered in critically ill patients. Sleep alterations involved both sleep duration and architecture and were consistent with previous reports [19, 26, 27], although the time spent in N3 sleep tended to be longer in our study. The severity of sleep alterations observed in this study shows that the study was conducted in patients with poor-quality sleep who were likely to benefit from an intervention designed to improve sleep quality. Of note, one of the strengths of our study was the use of polysomnography as a key outcome measure, because, to our knowledge, this study is one the largest studies including polysomnography recording in critically ill patients [4, 9, 19, 26–32]. However, performing polysomnography to provide analyzable data is a challenge in the ICU, as recently reported [33]. In contrast with recent reports, we did not observe the atypical sleep stages described in ICU patients, namely pathologic wakefulness and atypical sleep [29, 34], because the EEG patterns observed complied with the Rechtschaffen and Kales scoring system [22]. This result could be explained by the exclusion of patients in whom sedation or high-dose opioids (major sources of altered sleep and EEG patterns) [35] had been discontinued for < 24 h. It is also noteworthy that most patients in our study were not intubated at the time of polysomnography, although a substantial proportion of them had previously been intubated (Table 1) and were globally less severely ill than patients in previously published studies. This could explain the absence of atypical sleep recordings, which have been described mostly in critically ill patients [29, 34]. Of note, the intervention was applied from 10:00 p.m. to 8:00 a.m., which can be considered as too long. However, this allowed a little flexibility and guaranteed that patients would receive the intervention for at least 8 h. Sleep recording was performed from 2:00 p.m. to 8:00 a.m. in order to be consistent with most studies on sleep in the ICU. Also, we admit that sleep outside, and particularly before, the intervention is unlikely to be altered by the intervention. For this reason, we report our results for the whole duration of recording and also for nighttime.

The N3 percentage of total sleep time, which was the primary outcome of our study, was not significantly different between the two groups. Total sleep and night sleep times, as well as various descriptors of sleep architecture, were also not significantly different, suggesting that a protective strategy based on the use of earplugs and an eye mask at night does not improve sleep duration or architecture. These results are in contrast with those of previous studies reporting the benefit of this strategy. However, none of these studies were conducted in a general ICU population, and none were based on the use of polysomnography, because the only study that used polysomnography was conducted in healthy subjects subjected to ICU noise and light conditions [36]. In this study, earplugs and an eye mask improved REM sleep time and arousals but failed to improve N3 time, total sleep time, and sleep efficiency. Studies conducted in critically ill patients showed that earplugs and an eye mask were associated with an improvement in score-assessed sleep perception [12, 36, 37] as well as melatonin and cortisol levels [36]. In addition, a recent meta-analysis suggested that the use of earplugs and an eye mask was associated with a significant reduction of the incidence of delirium [38, 39]. None of these studies used polysomnography, and most of them were conducted not in a general ICU but in a postanesthesia care unit [12] or cardiac surgical ICU [36].

A major limitation of our study was that many patients did not wear earplugs and an eye mask all night long. Subsequently, the study was likely to be underpowered to detect a significant difference, as suggested by the increase in N3 stage sleep in the per-protocol analysis. Some patients removed their devices, whereas in others earplugs and the eye mask shifted during the night. The general tolerability of the intervention is a key to its success. Previous studies have shown that many patients found earplugs and even an eye mask uncomfortable or very uncomfortable [40], with compliance averaging 13% [38]. Patients complained about earplugs not staying in place and sore ears or reported feeling anxious when they did not hear any background noise [11, 40, 41]. Patients also complained that eye masks made them feel hot and sweaty and were too tight, causing a feeling of claustrophobia [40]. Consequently, patients may be unwilling to use earplugs or an eye mask [42]. This is all the more true in that patients of the intervention group who wore earplugs only part of night had even poorer sleep quality than patients in the control group. It raises the hypothesis that the poor tolerance of the device altered sleep architecture, possibly because of anxiety and claustrophobia. ICU staff should therefore improve the acceptability of these devices by clearly explaining the potential benefits to the patients, by helping patients to choose the best device in terms of shape and size, and by providing adequate assistance and instructions concerning their use [36]. Future improvement of the quality of these devices may also help to improve their tolerance.

Another limitation of our study that could have influenced the results was the fairly large proportion of N3 sleep in the control group. With a median of 11% of total sleep time, the proportion of N3 sleep observed in our study exceeded that reported in previous studies [9, 19]. This high proportion of N3 sleep could be consistent with the major efforts undertaken in our unit to improve sleep quality, including, but not limited to, lights turned off at 10:00 p.m. in rooms, televisions turned off at 10:00 p.m., and minimal room alarms. In addition, window blinds are raised during the daytime, and patient mobilization is encouraged in order to promote normal circadian rhythms. All patients are also accommodated in single rooms. Despite these measures, median sound levels were 55 (54–58) dB and 56 (54–57) dB in the control group and intervention group, respectively, with peaks > 70 dB. These levels are higher than those recommended by the World Health Organization (>35 dB) but similar to the noise levels reported in previous studies [43].

However, this protective strategy was associated with a reduction of prolonged awakenings, which suggests that earplugs and an eye mask had at least a minor impact on sleep architecture. It is of note that this is a relevant criterion in term of sleep disturbance and that one could argue that it would have been more logical to select it as the primary outcome because the main purpose of wearing earplugs and an eye mask is to prevent awakening by external stimuli. Nevertheless, in light of our results, it would be unreasonable to expect a single intervention, namely wearing earplugs and an eye mask, to dramatically improve sleep quantity and quality, which depend on multiple and complex determinants. Recently, Kamdar et al. reported the impact of a multifaceted intervention designed to improve sleep quality in ICU patients [8]. Earplugs and an eye mask were part of this strategy, which also included nighttime interventions to reduce sleep disruption at night and daytime interventions to promote a normal circadian rhythm. Although this multifaceted intervention did not significantly improve sleep quality, it did improve the perceived nighttime noise and the incidence of delirium [8]. In addition, although a correlation has been observed between noise level and arousal frequency in ICU patients [44], a questionnaire administered to patients after ICU discharge showed that other human interventions, such as phlebotomy and measurement of vital signs, were even more sleep-disruptive than noise [45]. Noise and care activities could account for < 15–30% of arousals and awakenings in ICU patients [9, 19].

## Conclusions

The use of earplugs and eye masks at night in ICU patents who have awoken from the effects of sedation did not increase the N3 proportion of sleep, but it did decrease the number of prolonged awakenings in ICU patients and also increased N3 duration when these devices were well tolerated. Earplugs and an eye mask had no impact on outcome. A major limitation of this study is the limited willingness of patients to use these devices, which were the source of numerous complaints. It seems that the benefit of wearing earplugs and an eye mask is at least partially counteracted by the discomfort of wearing the devices. Because the improvement of sleep quality in ICU patients remains a major concern, future studies should evaluate multifaceted programs, possibly including protective devices, rather than focusing on a single intervention.

## Additional files

Additional file 1: Table S1. Additional patient characteristics prior to inclusion. (DOC 30 kb)

Additional file 2: Table S2. Nurse interventions and noise level. (DOC 33 kb)

Additional file 3: Table S3. Main sleep characteristics in patient subgroups according to whether patients wore earplugs all night long. (DOC 33 kb)

Additional file 4: Figure S1. Study flowchart including patients followed at ICU discharge, hospital discharge, and 90 days after inclusion. (DOC 102 kb)

## Abbreviations

EEG: Electroencephalography
HADS: Hospital Anxiety and Depression Scale
ICU: Intensive care unit
IES-R: Impact of Event Scale–Revised
RASS: Richmond Agitation-Sedation Scale
REM: Rapid eye movement
SAPS: Simplified Acute Physiology Score
TST: Total sleep time
VAS: Visual analogue scale
