# Supplementary material for: Impact of earplugs and eye mask on sleep in critically ill patients: a prospective randomized study
Source: Crit Care. 2017 Nov 21;21:284. doi: 10.1186/s13054-017-1865-0 (PMC5696771; doi:10.1186/s13054-017-1865-0)
Supplement: Supplementary file 1 — Additional patient characteristics prior to inclusion. (DOC 30 kb) [file 13054_2017_1865_MOESM1_ESM.doc]

**Table S1. Additional patient characteristics prior to inclusion**

|  | **Control**  **Group**  n=31 | **Intervention**  **Group**  n=30 | **p** |
| --- | --- | --- | --- |
| **Sedative treatments, *n (%)***  Benzodiazepines  Clonazepam  Diazepam  Bromazepam  Midazolam  Opioids  Morphine  Sufentanil  Propofol  Others  Ketamine  Zolpidem, Zopiclone  Hydroxyzine  Haloperidol | 21 (68)  9  0  1  0  8  13 (42)  5  8  6 (19)  3 (10)  1  1  0  1 | 17 (57)  10  1  1  1  7  14 (47)  5  9  5 (17)  8 (27)  5  1  1  1 | 0.30 |
| **Days of sedation**  Benzodiazepines  Opioids  Propofol  Other | 3 (1-5)  3 (1-8)  1 (0-2)  0 (0-1) | 1 (1-3)  2 (1-6)  3 (1-3)  1 (0-2) |  |
| **Vasoactive treatments, *n (%)***  Norepinephrine  Epinephrine | 9 (29)  8  1 | 6 (20)  4  2 | 0.41 |

Results are expressed as median (interquartile range) or frequency (%).
