# Supplementary material for: Impact of earplugs and eye mask on sleep in critically ill patients: a prospective randomized study
Source: Crit Care. 2017 Nov 21;21:284. doi: 10.1186/s13054-017-1865-0 (PMC5696771; doi:10.1186/s13054-017-1865-0)
Supplement: Supplementary file 2 — Nurse interventions and noise level. (DOC 33 kb) [file 13054_2017_1865_MOESM2_ESM.doc]

**Table S2. Nurse interventions and noise level**

|  | **Control**  **group**  n=31 | **Intervention group**  n=30 | ***p*** |
| --- | --- | --- | --- |
| ***From 3:00 pm to 10:00 pm*** | | | |
| Mean noise level, *dB* | 55 (54-58) | 56 (54-57) | 0.87 |
| Maximum noise level, *dB* | 86 (83-91) | 86 (82-92) | 0.74 |
| Time with >70 dB, *min* | 3 (1-6) | 3 (1-6) | 0.78 |
| ***From 10:00 pm to 8:00 am*** | | | |
| Mean noise level, *dB* | 52 (51-54) | 52 (51-53) | 0.94 |
| Maximum noise level, *dB* | 84 (81-89) | 82 (78-87) | 0.09 |
| Time with >70 dB, *min* | 1 (0-4) | 1 (0-4) | 0.62 |
| ***Nurse interventions*** | | | |
| Total duration, *min* | 31 (25-57) | 25 (15-48) | 0.39 |
| Intervention time with lights on during nighttime ^a^, *min* | 25 (14-50) | 14 (10-40) | 0.17 |
| Number of interventions with lights on during nighttime ^a^, *n* | 3 (3-5) | 2 (1-3) | 0.02 |

dB, decibel.

Results are expressed as median (interquartile range).

^a^Nighttime is 10:00 pm to 8:00 am.
