# Supplementary material for: Impact of earplugs and eye mask on sleep in critically ill patients: a prospective randomized study
Source: Crit Care. 2017 Nov 21;21:284. doi: 10.1186/s13054-017-1865-0 (PMC5696771; doi:10.1186/s13054-017-1865-0)
Supplement: Supplementary file 3 — Main sleep characteristics in patient subgroups according to whether patients wore earplugs all night long. (DOC 33 kb) [file 13054_2017_1865_MOESM3_ESM.doc]

**Table S3. Main sleep characteristics in patient subgroups according to whether or not patients wore earplugs all night long**

|  | **Control**  **Group**  n=28 | **Wore earplugs**  **All night long**  n=15 | **p** |
| --- | --- | --- | --- |
| Total sleep time *^a^, min* | 301 (221-402) | 387 (215-447) | 0.352 |
| Total sleep time during nighttime *^b^*, *min* | 274 (177-329) | 335 (206-404) | 0.214 |
| N3 Stage, *min* | 31 (7-76) | 74 (32-106) | 0.039 |
| N3 Stage, *% of total sleep time* | 11 (3-23) | 22 (6-33) | 0.071 |
| REM sleep, *min* | 32 (5-49) | 45 (9-88) | 0.261 |
| Sleep efficiency, *% per 18 h* | 27 (20-39) | 38 (21-43) | 0.532 |
| Night sleep efficiency, *% per 10 h* | 47 (32-61) | 58 (41-68) | 0.178 |
| Short awakenings (<1min), *n* | 12 (5-20) | 9 (3-13) | 0.352 |
| Long awakenings (≥1 min), *n* | 31 (21-50) | 17 (13-26) | 0.010 |
| Self-assessed sleep quality, *VAS* | 50 (32-70) | 50 (45-70) | 0.999 |

REM, rapid eye movement; VAS, visual analog scale from zero (poor sleep quality) to 100 (excellent sleep quality).

^a^Total recording period is 2:00 pm to 8:00 am.

^b^Nighttime is 10:00 pm to 8:00 am.
