# Supplementary material for: Impact of earplugs and eye mask on sleep in critically ill patients: a prospective randomized study
Source: Crit Care. 2017 Nov 21;21:284. doi: 10.1186/s13054-017-1865-0 (PMC5696771; doi:10.1186/s13054-017-1865-0)

**Figure S1. Study flow chart including patients followed at ICU discharge, hospital discharge and 90 days after inclusion.**


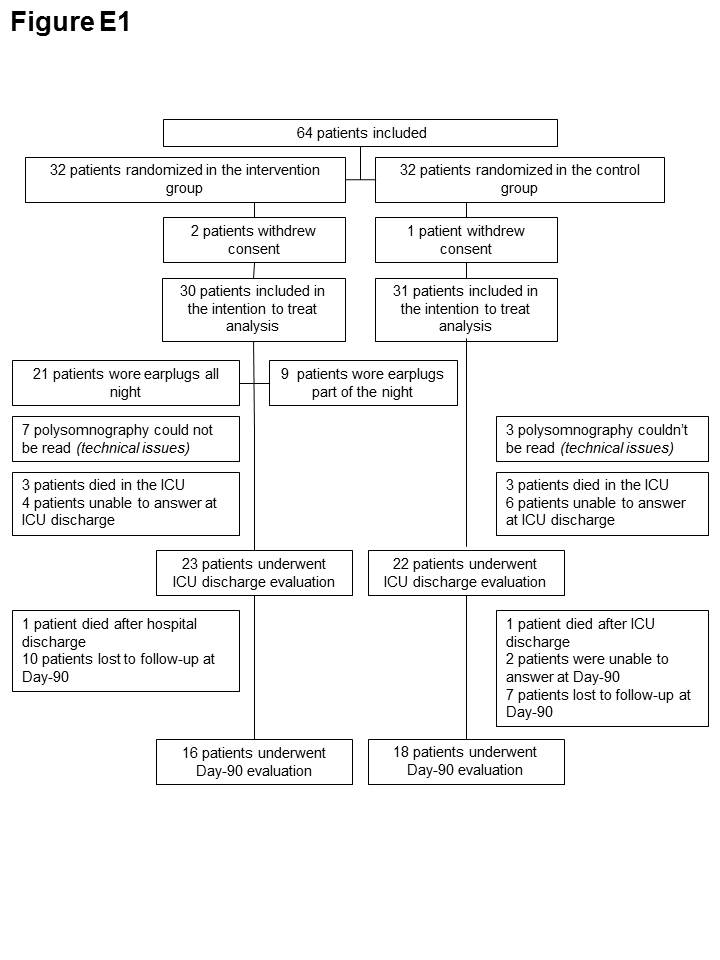

Supplement: Supplementary file 4 — Study flowchart including patients followed at ICU discharge, hospital discharge, and 90 days after inclusion. (DOC 102 kb) [file 13054_2017_1865_MOESM4_ESM.doc]
